# DROMPA: easy-to-handle peak calling and visualization software for the computational analysis and validation of ChIP-seq data

**DOI:** 10.1111/gtc.12058

**Published:** 2013-05-15

**Authors:** Ryuichiro Nakato, Tahehiko Itoh, Katsuhiko Shirahige

**Affiliations:** 1 Research Center for Epigenetic Disease, Institute of Molecular and Cellular Biosciences, The University of TokyoTokyo, 113-0032, Japan; 2 CREST, JST, K's Gobancho7 Gobancho, Chiyoda-ku, Tokyo, 102-0076, Japan; 3 School and Graduate School of Bioscience and Biotechnology and Bio-Frontier Research Center, Tokyo Institute of TechnologyYokohama, 226-8501, Japan

## Abstract

Chromatin immunoprecipitation with high-throughput sequencing (ChIP-seq) can identify genomic regions that bind proteins involved in various chromosomal functions. Although the development of next-generation sequencers offers the technology needed to identify these protein-binding sites, the analysis can be computationally challenging because sequencing data sometimes consist of >100 million reads/sample. Herein, we describe a cost-effective and time-efficient protocol that is generally applicable to ChIP-seq analysis; this protocol uses a novel peak-calling program termed DROMPA to identify peaks and an additional program, parse2wig, to preprocess read-map files. This two-step procedure drastically reduces computational time and memory requirements compared with other programs. DROMPA enables the identification of protein localization sites in repetitive sequences and efficiently identifies both broad and sharp protein localization peaks. Specifically, DROMPA outputs a protein-binding profile map in pdf or png format, which can be easily manipulated by users who have a limited background in bioinformatics.

## Introduction

Identification of protein-binding sites in a genome can be achieved using chromatin immunoprecipitation with high-throughput sequencing (ChIP-seq) to clarify the biological role(s) of targeted proteins (Park 2009). With advances in high-throughput DNA sequencing technologies, it has become possible to perform large-scale ChIP-seq studies that allow comparison of a considerable number of samples. For example, in a recent study, nearly 200 human ChIP samples were processed in parallel (Ernst *et al*. 2011). As one human ChIP-seq sample contains approximately 20 Gb (gigabases) of short read sequences, there is a clear demand for a protocol that can efficiently analyze a large amount of ChIP-seq data in a short period.

**Figure 1:**
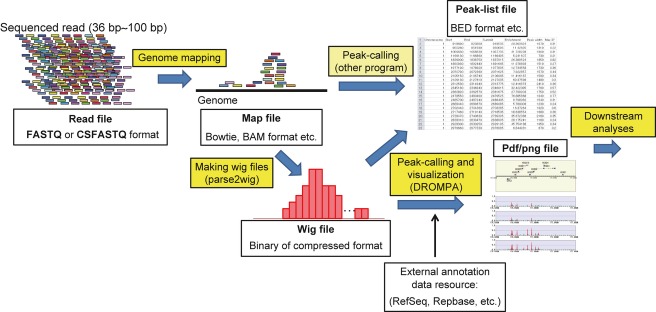
Workflow for ChIP-seq analysis. Immunoprecipitated DNA is sequenced and used to generate a FASTQ- (Illumina) or csfasta/qual-formatted (SOLiD) read file. The read file is mapped onto the reference genome sequence by a mapping tool. Parse2wig converts this map file into a wig file for each chromosome, and DROMPA implements peak calling and visualization using the wig files. The peak list is outputted in a tab-delimited text file, which can be used as a bed file. The visualization data are outputted in pdf or png format with genomic annotation data specified. Compressed wig files generated by parse2wig can be used for other visualization programs and browsers.

ChIP-seq analysis can be divided into three steps (Fig. 1). (i) Genome mapping: after acquiring the sequence data, the sequences are mapped onto a reference genome sequence to generate a map file that contains information about the location of each sequence (read). (ii) Peak calling: identification of regions in which reads are significantly enriched compared with the read distributions in a control (input) sample. (iii) Characterization of the protein-binding sites: identification of DNA sequence motifs shared among the binding sites of the targeted protein or correlation of binding sites with other -omics data (e.g., other protein-binding profiles), DNA methylation data or gene expression data to find functional links to biological processes.

For species with small genome sizes (approximately 20 Mbp), such as *Saccharomyces cerevisiae* or *Schizosaccharomyces pombe*, a greater than 10-fold sequencing depth in both the input and the ChIP fraction can be easily obtained with relatively low sequencing cost. Given enough sequencing depth in the input fraction and an equivalent number of sequence reads in the ChIP fraction, tuning the values of threshold parameters for peak calling is not troublesome. As the statistical reliability at every genomic position is large in each case, the protein-binding profile map (the ratio of sequence reads from the ChIP fraction and the input fraction, which is calculated for a running window of fixed size and plotted against the genome position on a linear or log scale) reflects the actual protein-binding probability at every position in the genome (Hu *et al*. 2011; De Piccoli *et al*. 2012; Enervald *et al*. 2013). However, even with a dramatic increase in the number of sequence reads per run provided by next-generation sequencers, it is not realistic to cover the equivalent of one billion sequence reads or more to get >10-fold sequencing depth for species with large genome sizes (i.e. genomes containing more than 3 Gbp). Consequently, when handling human or mouse ChIP-seq data, threshold parameters for peak calling need to be set by trial and error, which can be time-consuming and difficult.

Quick visualization of ChIP-seq results, when presented as a ChIP read distribution or protein-binding profile map, is an invaluable means for rapidly assessing data quality and setting threshold parameters for peak calling. As available visualization protocols require installation of additional software, which may require time and effort to manage, it is difficult to show the whole-genome distribution of the mapped reads and detected peaks to a user who has little bioinformatics skill. As an alternative means of visualization, the data can be uploaded to the UCSC genome browser (http://genome.ucsc.edu/) to visualize the read distributions and to incorporate various genomic annotations into the data file. However, there are several disadvantages to using a web-based system when the data set is large. First, uploading a huge data set is time-consuming. Second, there is a limitation in showing multiple sets of ChIP-seq data for different projects separately. Third, the uploaded data are discarded when the user does not access the site for a given period (e.g., 48 h for the UCSC genome browser unless the user creates an account in UCSC).

Herein, we present a time-efficient and cost-effective computational protocol that incorporates our novel program DROMPA (DRaw and Observe Multiple enrichment Profiles and Annotation) for peak calling and visualization. We also developed a program named parse2wig for preprocessing map files into wig files. We validated our programs using 29 samples from three human cell lines (HeLa cells, B cells and fibroblasts) in a study of HDAC8 mutations in Cornelia de Lange syndrome DNA using antibodies directed against Rad21, Smc3ac and CTCF (Deardorff *et al*. 2012). DROMPA accurately identified the binding sites of these proteins with a low false discovery rate (FDR) of <0.3% for almost all ChIP samples analyzed. Our workflow has also been applied to ChIP-seq analyses of the genomes of *S. cerevisia*e (Mishra *et al*. 2010; Hu *et al*. 2011; Kegel *et al*. 2011; Kurze *et al*. 2011; Tanaka *et al*. 2011; De Piccoli *et al*. 2012; Enervald *et al*. 2013), *S. pomb*e (Tazumi *et al*. 2012), human (Deardorff *et al*. 2012) and mouse (Yamaji *et al*. 2013). Below, we describe the workflow of our protocol using these programs and compare its performance with several other available programs. DROMPA and its associated program parse2wig require much less computation memory and time to achieve similar sensitivity and specificity. We also discuss how best to apply the workflow and assess data quality.

The software package and several annotation files for DROMPA are available on the website (http://www.iam.u-tokyo.ac.jp/chromosomeinformatics/rnakato/drompa/, see Appendix S1 in Supporting Information).

## Results

### Overview

Figure 1 shows the workflow for our ChIP-seq procedure described in the Introduction. Using parse2wig, the map file is first converted into a wig file for each chromosome (Fig. 1, ‘Making wig files’). These files contain information about how many reads are mapped to each genomic region (‘bin’) and are used as input for peak calling and visualization by DROMPA. Parse2wig also filters out bias introduced by PCR amplification (Kozarewa *et al*. 2009) and normalizes the number of reads in each bin. Taking the wig file data as input, DROMPA identifies peaks and outputs bar graphs for the read distributions and/or ChIP/control enrichment profiles.

DROMPA calls peaks by comparing the read distribution of the ChIP sample with that of the corresponding input sample (see Experimental Procedures). The distribution of mapped reads is affected by various genomic features (proportion of unique and repetitive sequence and copy number variation), by the methods for ChIP and library construction and by platform-specific sequencing efficiency (Dohm *et al*. 2008; Liu *et al*. 2010; Landt *et al*. 2012). Consequently, it has been suggested that the input might have several ‘pseudobinding sites’ that associate with actively transcribed promoter regions (Auerbach *et al*. 2009). Filtering out such pseudobinding sites is only possible by comparing the ChIP sample with an input sample, and this step is essential for the specificity of peak calling (Rozowsky *et al*. 2009). We therefore strongly recommend that the ChIP data be compared with the corresponding input data to decrease identification of false-positive protein-binding sites. There are also several programs that can detect peaks without the inclusion of an input sample.

DROMPA can accept multiple mapped reads (reads mapped on multiple loci of the reference genome), whereas most available peak-calling programs can only accept uniquely mapped reads (reads mapped onto the reference genome only once). This feature is clearly important for identifying peaks in repetitive sequences. Please refer to Experimental and Appendix S1 (Supporting Information) for detailed information on algorithm and experimental design.

### Visualization of ChIP-seq data by DROMPA

**Figure 2:**
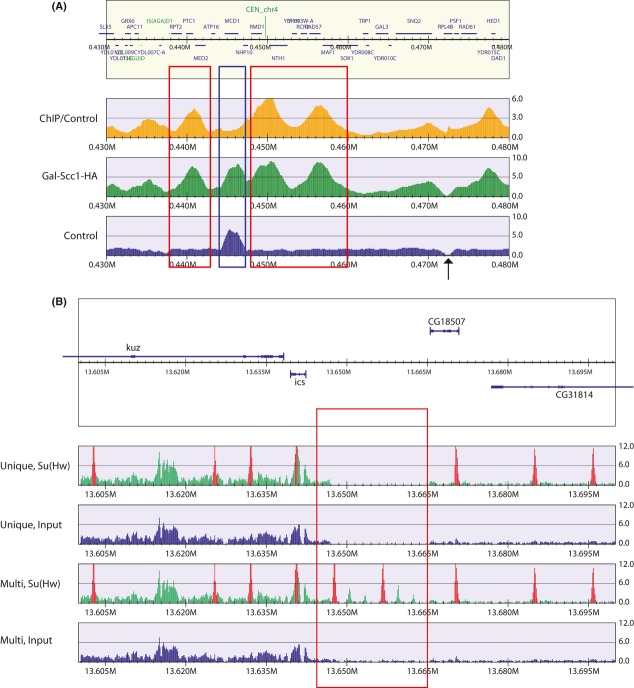
Examples of visualization and analysis by DROMPA. We reanalyzed (a) *Saccharomyces cerevisiae* Scc1 ChIP-seq data from Enervald *et al*. (2013) and (b) *D*. *melanogaster* Suppressor of Hairy-wing, Su(Hw), ChIP-seq data from Chen *et al*. (2012) using DROMPA. The orange, green and blue histograms represent ChIP/control ratio, ChIP read distributions and input-read distributions, respectively. The *y*-axis values of the read distribution histograms are the normalized ChIP read intensities (*R*_x_) for each bin (see Experimental Procedures). (a) Part of *S*. *cerevisiae* chromosome II (nucleotide numbers 230–300 kbp) with *Saccharomyces* Genome Database annotation. The red boxes and blue box indicate true and pseudobinding sites, respectively. The black arrow indicates a region with few mapped reads. (b) Part of *Drosophila melanogaster* chromosome 2L (build dm3, nucleotide numbers 13.6–13.7 m) with RefSeq gene annotation. In the gene annotation, the thick lines indicate DNA exons and the thin lines indicate DNA introns. For the top two panels, reads uniquely mapped to the genome were used. For the bottom two panels, both multiply and uniquely mapped reads were used. Regions in which reads were significantly enriched are in red.

The ChIP-seq data for Scc1 of *S. cerevisiae* (Enervald *et al*. 2013) visualized by DROMPA are shown in Fig. 2a. The profile of ChIP/input ratio effectively identifies the ChIP-enriched regions (red boxes) and filters out the false-positive peak (blue box) and the low-coverage region (black arrow). To examine the effectiveness of using multiple mapped reads, we used ChIP-seq data of *Drosophila melanogaster* transcription factor suppressor of Hairy-wing [Su (Hw)] (Chen *et al*. 2012). Chen *et al*. showed that approximately 1% of ChIP-chip peaks was not detected by ChIP-seq even with deep sequencing (corresponding to approximately 327 m reads in humans), due to the low mappability of uniquely mapped reads in several genomic regions. By allowing multiple mapped reads, peaks can be identified from such regions (Fig. 2b).

**Figure 3:**
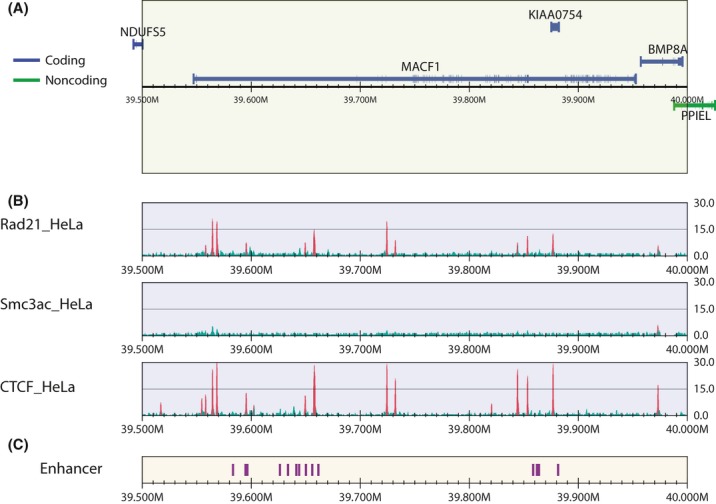
Visualization of the Rad21, Smc3ac and CTCF-binding sites on the MACF1 gene by DROMPA. (a) The regions including and surrounding the MACF1 gene on HeLa cell chromosome 1 (human genome build hg19, nucleotide numbers 39.5–40.0 m) with RefSeq gene annotation. In the gene annotation, the thick lines indicate DNA exons and the thin lines indicate DNA introns. ‘Coding’ or ‘noncoding’ means that the prefix of the accession number is ‘NM’ or other. (b) Rad21 (top), Smc3ac (middle) and CTCF (lower) read distributions for the MACF1 gene and surrounding regions. The *y*-axis values are the normalized ChIP read intensities (*R*_x_) for each bin. The control read distributions and/or the ChIP/control enrichment profiles can also be plotted if desired. Regions in which reads were significantly enriched are in red. (c) The enhancer regions found by Heintzman *et al*. (2009) are shown as purple bars.

**Figure 4:**
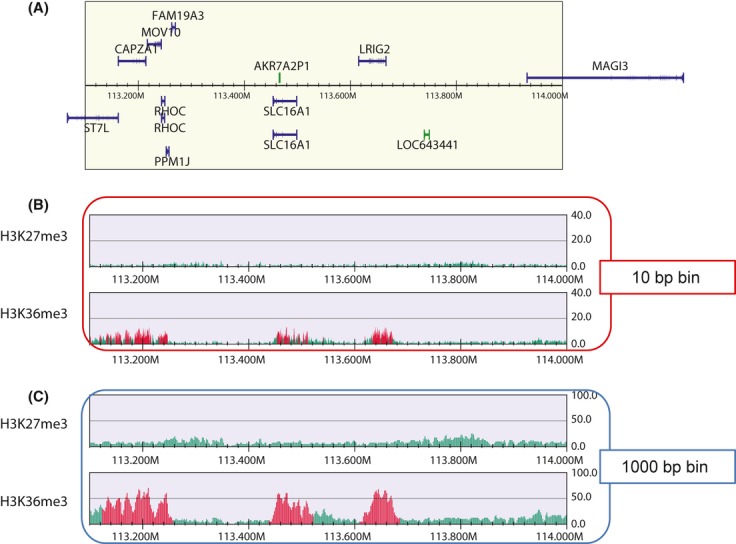
Detection of broad peaks by DROMPA (a) Part of HeLa cell chromosome 1 (human genome build hg19, nucleotide numbers 113.1–114.0 m) with RefSeq gene annotation. In the gene annotation, the thick lines indicate DNA exons and the thin lines indicate DNA introns. (b) The read distributions for H3K27me3 (upper panel) and H3K36me3 (lower panel) using 10-bp bins and a smoothing width of 500 bp. Regions in which reads were significantly enriched are in red. These parameters are appropriate when sharp peaks (approximately 1 kb) are expected (e.g., for transcriptional factor–related peaks). (c) The read distributions of H3K27me3 and H3K36me3 using 1-kbp bins and a smoothing width of 2 kbp. Regions in which reads were significantly enriched are in red. These parameters are appropriate when broader peaks (10–100 kbp) are expected. A comparison of panels (b) and (c) shows that because H3K27me3 and H3K36me3 bind over a broad DNA region, the enriched regions cannot be detected properly if the parameter set for calling of sharp peaks is applied. However, application of a 1-kbp bin size allows the enriched regions for each histone modification to be distinguished.

**Figure 5:**
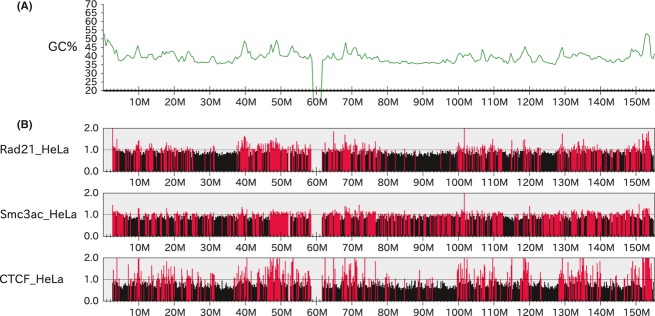
Chromosome-wide visualization. Chromosome-wide visualization offers a macroscopic view of protein-binding profiles. (a) GC contents with 500-kp windows are plotted. (b) Macroscale protein-binding profiles for the human X chromosome were generated using DROMPA with the ‘-wg’ option and a 100-kbp bin. In this figure, ‘-scale_ratio’ is set to 1, and bins in which ChIP/control >1 are highlighted in red and others in black. The region in which the enrichment value is 0 (i.e. 59–61 Mbp) is one for which no sequence information is available.

Examples of visualizations for human HeLa cells are shown in Fig. 3 [for a typical transcriptional factor–related binding site with the published enhancer annotation (Heintzman *et al*. 2009)], Fig. 4 (for histone modifications, which have broad peak distribution) and Fig. 5 (for a chromosome-wide read enrichment distribution). As previously described (Park 2009), an appropriate parameter set for peak calling depends on the binding mode of the target protein. When broader peaks (10–100 kbp) are expected, it is necessary to enlarge the bin size and relax the threshold value for each parameter. When the binding mode of the protein is unclear, we recommend trying the parameter sets for sharp and broad peaks. Even if no peaks are detected, chromosome-wide visualization may help to identify preferential protein localization regions.

### Performance comparison

To assess the performance of DROMPA, we used our previously published ChIP-seq data for human Rad21, acetylated Smc3 (Smc3-ac) and CTCF (Deardorff *et al*. 2012). A detailed description of the data analyses is provided in the Experimental Procedures. We compared the results provided by our programs with those of MACS (Zhang *et al*. 2008), PeakSeq (Rozowsky *et al*. 2009) and Quest (Jothi *et al*. 2008), the most widely used peak-calling programs and which use different peak-calling algorithms to that of DROMPA.

The computation time and memory requirements of the various programs are summarized Table 1. For brevity, we show the results for DROMPA and parse2wig when binary or compressed wig files are used. DROMPA in combination with binary wig files provided the fastest computation time, and memory requirements were an order of magnitude smaller than those of the other programs. When multiple ChIP-control pairs were used, DROMPA was more memory efficient compared with separate analysis of each pair (Fig. S1 in Supporting Information).

**Table 1:** Computation time and memory required for each program

	Time (s)			Memory (MB)		
		
	Rad21	Smc3ac	CTCF	Rad21	Smc3ac	CTCF
Peak calling						
MACS	1161.17	1282.13	944.15	3671	4688	3143
PeakSeq	300.88	439.19	193.71	4218	4999	2899
Quest*	1463.24	1452.58	1953.15	9947	10283	10 000
DROMPA (binary)	94.39	90.34	98.54	382	429	429
DROMPA (compressed)	567.58	585.12	506.17			
Preprocessing						
PeakSeq preprocess	148.69	237.46	84.52	393	612	280
Parse2wig (binary)	71.74	108.01	44.68	785	1025	749
Parse2wig (compressed)	235.47	296.9	158.1			

* We summed the memory used for two ‘QuEST_align_2_b’ program runs, which were executed simultaneously.

The disk space required for each data format is summarized in Table S1 (Supporting Information). As the sizes of the binary and compressed wig files are smaller than those of the map file, the disk storage space required for the ChIP-seq analysis can be decreased by retaining only the wig files because DROMPA requires only wig files as input. Although PeakSeq also preprocesses a map file, the size of the preprocessed data was larger than the total size of the wig files generated by parse2wig. Consequently, DROMPA efficiently saves disk space compared with other programs.

### Sensitivity and specificity of DROMPA for peak detection

**Figure 6:**
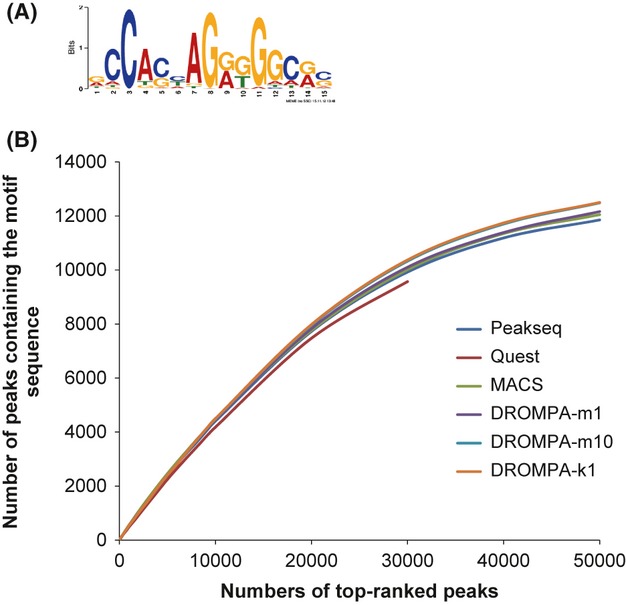
Performance test for the identification of the canonical CTCF-binding site motif. (a) The reference motif sequence used for the performance test. (b) The number of peaks containing the motif sequence (*y*-axis) was plotted against increasing numbers of top-ranked peaks returned by each program (*x*-axis).

To assess the sensitivity and specificity of the programs, we used a list of high-scoring motif sites extracted from the whole-genome sequence of the reference binding-motif sequence for CTCF. To extract this canonical motif sequence, we obtained reference CTCF-binding site data from Rhee & Pugh (2011) and used only binding-site sequences that were identified in all three biological replicates. We used MEME (Bailey & Elkan 1994), with a *P*-value threshold of <10^−6^, to extract the canonical motif sequence shown in Fig. 6a. We then searched for genomic regions that possessed the canonical motif sequence using MAST (Bailey & Gribskov 1998) with a *P*-value threshold value of <10^−6^. In total, 18,289 motif sites were obtained from the whole-genome sequence. To compare the sensitivities and specificities of the programs, we selected the top-ranked peaks returned by each program and analyzed the number of peaks that contained the canonical motif sequence, in a manner similar to that used by. Boeva *et al*. (2010). For DROMPA, we used three mapping parameter options: ‘-m1’ (uniquely mapped reads), ‘-m10’ (all mapped loci per read mapped ≤10 times) and ‘-k1’ (best matched locus per all multiple mapped reads). We used uniquely mapped reads for the analysis by DROMPA and found that the number of identified peaks containing the canonical motif of CTCF was similar to the number of peaks identified by MACS or PeakSeq (Fig. 6b). We also observed an increase in sensitivity when we used both multiple and uniquely mapped reads for the analysis by DROMPA. This is possibly due to increases in the sequencing depth and in the proportion of accessible regions (Chung *et al*. 2011).

### Sample quality assessment

The total number of mapped reads is an important issue when evaluating the quality of ChIP-seq data. A large percentage of unmapped reads (>50%) generally indicates that the sample DNA is of poor quality (e.g., ChIP efficiency is low or the DNA sample is contaminated by DNA from other species). Even if the number of sequenced reads is large, the mapping ratio (the number of mapped reads/the number of sequenced reads) may be poor if the initial amount of DNA is insufficient, for example, because of inefficient ChIP. In this situation, the number of mapped reads will not be sufficient for statistical analysis: for example, when 20 million reads are sequenced and the mapping ratio is 15%, only 3 million reads are available for analysis. The mapping results obtained here for the test data are summarized in Table 2. The high percentage of mapped reads is a guarantee of the high quality of the sequenced samples. The percentage of uniquely mapped reads was approximately 60% for all samples, which is empirically considered to be a good result for a SOLiD sequencer. For an Illumina sequencer, the average percentage of uniquely mapped reads is generally >80%. When allowing multiple mapped reads, the number of available reads increases, which allows the number of repetitive reads to more greatly influence the results. If the DNA sample is PCR-amplified >20 times, then although the number of mapped reads will be sufficient, the number of reads that are filtered out as biased may also be large; this may lead to a substantial number of reads that cannot be used further. Therefore, the PCR bias percentage indicates the redundancy of the sequenced reads. A similar suggestion was made previously by Landt *et al*. (2012). Usually the PCR bias percentage is <20% of the total reads. However, when micrococcal nuclease is used to shear DNA, the PCR bias percentage can increase to approximately 30%. If the PCR bias percentage is >40%, over-amplification may occur; if it is >80%, it may be difficult to accurately identify peaks. Ideally, >30 × 10^6^ unique reads should be mapped with <20% having to be filtered out as PCR-biased reads. In our previous work (Deardorff *et al*. 2012), we used 3–7 × 10^7^ uniquely mapped reads for each human sample.

**Table 2:** Mapping results of test data onto human genome build hg19

Sample	Total reads	Uniquely mapped reads	%	Reads mapped to multiple loci*	%	Unmapped reads	%	Reads filtered as PCR bias	%	Reads remaining after filtering	%
Rad21 (HeLa)	76 354 774	49 710 285	65.10	7 472 860	9.79	19 171 629	25.11	5 329 568	6.98	44 380 717	58.12
Smc3ac (HeLa)	121 365 117	76 858 790	63.33	11 892 522	9.80	32 613 805	26.87	15 364 647	12.66	61 494 143	50.67
CTCF (HeLa)	42 742 350	28 754 522	67.27	3 736 358	8.74	10 251 470	23.98	4 359 289	10.20	24 395 233	57.08
Control (HeLa)	111 252 274	67 341 466	60.53	9 881 093	8.88	3 402 971	3.06	5 822 912	5.23	61 518 554	55.30

* When using multiple mapped reads, the number of usable reads increases by approximately 10%.

### Peak assessment

**Figure 7:**
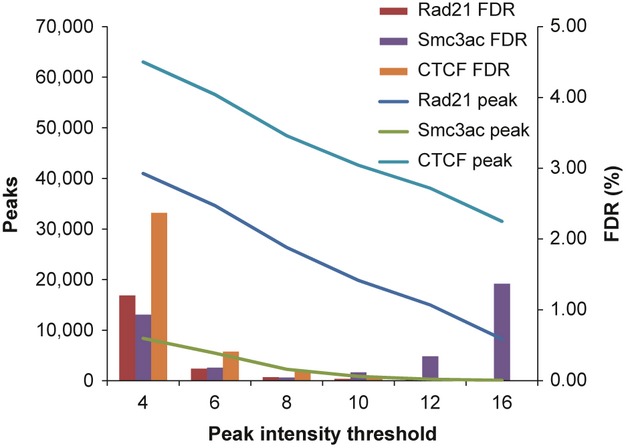
Peak numbers and FDRs obtained when different peak-intensity threshold values were used. When specifying a more stringent threshold value, the number of detected peaks decreases. For Smc3ac, when the threshold value was set to 16, the FDR increased relative to that of a threshold of 8, despite the decrease in the number of control peaks. This increase occurred because the number of ChIP peaks was too small to enable accurate calculation of the FDR. Lines, peak number; bars, FDR.

The number of peaks detected and the FDRs for the Rad21, Smc3-ac and CTCF samples are given in Table S2 (Supporting Information). A small FDR indicates that the threshold value for peak calling is sufficiently stringent. The correlations of number of peaks detected and FDRs at different peak-intensity threshold values are shown in Fig. 7. The intensities of most control peaks were small; thus, as the peak threshold value became more stringent, the number of control peaks decreased more rapidly than did the number of ChIP peaks, which improved the FDR value. For Smc3-ac, however, when the number of ChIP peaks was too small, the FDR value increased. This result indicates that FDR does not work well under some circumstances, such as when the number of ChIP peaks is too small. It is important to check mapped read distributions and identified peaks by validating positive and negative binding sites by ChIP-qPCR.

## Discussion

In conclusion, the programs we have developed, DROMPA and parse2wig, offer the following advantages over other ChIP-seq analysis programs:

- Our programs require much less memory and time than other available peak-calling programs (reviewed by Laajala *et al*. 2009), making it possible to analyze large-scale ChIP-seq data (e.g., more than 10 human samples and/or multiple executions of each sample with trial-and-error determination of threshold levels) on a conventional desktop computer.
- DROMPA outputs protein-binding profiles and identifies peaks as bar graphs in a pdf or png format, both of which are compatible with many computer platforms and can be handled without installation of an additional visualization program. If the user wants to quickly access the ChIP-seq results to judge whether the experiment was successful or to share the results with collaborators elsewhere, DROMPA output is an efficient way to do so. Furthermore, pdf files are accepted by many scientific journals and easily processed by all available graphic software.
- DROMPA identifies protein-binding sites when the peaks are sharp (approximately 1 kbp, e.g., transcriptional factor binding sites) and when they are broad (approximately 1 Mbp, e.g., modified histone binding sites, such as H3K27me3 and H3K36me3), as a consequence of the simple peak-calling strategy used.
- DROMPA accepts multiple mapped reads, whereas most available peak-calling programs can only use uniquely mapped reads as input. Incorporation of multiple mapped reads into the calculation improves sequencing depth (up to 25%) (Chung *et al*. 2011) and helps identify binding sites in regions containing repetitive sequences.
- Our programs can be used for any species for which a genome sequence is available, that is, *S*. *cerevisiae*, *S. pombe, D. melanogaster,* chicken, mouse and human, among many others.

As shown in the performance comparison, DROMPA is the fastest program and is an order of magnitude more memory efficient than other programs. Preprocessing a map file and storing chromosome-separated wig files allows DROMPA to reduce the computation time for parsing a map file and to require memory for only one chromosome, which reduces the consumed memory for peak calling. PeakSeq also uses a preprocessing strategy, but also uses whole-genome data simultaneously on peak calling, which results in heavy consumption of memory. We also showed that DROMPA has similar sensitivity and specificity with other available programs using human ChIP-seq data. When allowing multiple mapped reads in addition to uniquely mapped reads, the sensitivity improved because of the expansion of accessible regions in the genome (Fig. 2b).

It is extremely difficult to evaluate the accuracy of peak calling because of the lack of ‘true’ binding-site data. As the optimal parameter set depends on the characteristics of the samples and there is no consistent threshold across different conditions for peak calling, then investigation of a protein whose binding mode is unknown necessitates the setting of threshold parameters for peak calling by trial and error. Another assessment for peak quality, the irreproducible discovery rate (IDR) methodology (Li *et al*. 2011), has been developed to assess replicate agreement and set thresholds. However, when the quality of one replicate is poor, possible true peaks in higher-quality replicates will be filtered (Landt *et al*. 2012). In this study, we focused on a canonical motif analysis of CTCF and showed a characteristic region for *Drosophila*. In future, we intend to improve DROMPA so that it can be applied to even relatively low-quality data with no biological replicates, without trial and error.

## Experimental procedures

### Data sets

The source codes for DROMPA and parse2wig are available on the DROMPA website (http://www.iam.u-tokyo.ac.jp/chromosomeinformatics/rnakato/drompa/). In this study, we used DROMPA version 1.1.1. Check the website for the latest version.

The ChIP-seq data were downloaded from The Sequence Read Archive (SRA, http://www.ncbi.nlm.nih.gov/sra) under accession number SRA062112 (*S*. *cerevisiae* Gal-Scc1-HA), SRP005957 (*D. melanogaster* Suppressor of Hairy-wing), SRP006944 (HeLa cell H3K27me3 and H3K36me3) and SRP011927 (HeLa cell Rad21, Smc3-ac and CTCF).

### Parse2wig: Converting mapped reads into wig data

Parse2wig sums the number of mapped reads in a bin (default value 10 bp) sequentially along a chromosome and outputs a wig-formatted file for each chromosome. Each mapped read is extended to an average, predetermined fragment length (default value 150 bp) as previously described (Chung *et al*. 2011; Rozowsky *et al*. 2009). Parse2wig can also be used with multiple mapped reads, which are divided equally among all locations (each mapped locus is weighted equally). Thus, the total number of reads mapped into bin *x* is 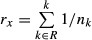 where *n*_k_ is the number of times that read *k* is mapped onto the reference genome and *R* is the full set of reads mapped in bin *x*.

Obviously, over-amplified templates reduce the accuracy of the statistical analysis, and the sequence reads derived from those templates must be filtered out as PCR bias before statistical analyses. To achieve this, redundantly mapped reads (reads starting exactly at the same 5’-sequence ends) over *T*_b_ times are filtered out as PCR bias. A similar strategy was proposed previously as a ‘nonredundant fraction’ (Landt *et al*. 2012), which simply set *T*_b_ > 1. For DROMPA, *T*_b_ is set as the larger of >1 and >*E*(r) × 10, where *E*(r) is the expected value of a mapped read for each base in the strand *s*, that is, *E*(*s*) = *N*^s^/*L* where *N*^*s*^ is the number of reads mapped on the strand *s* of the genome and *L* is the genome length. A similar strategy has been used for programs developed for genome mapping and repeat masking (Gotoh 2008; Morgulis *et al*. 2006).

To compare the results of multiple ChIP experiments, parse2wig uses a modified RPKM (reads per kilobase of exon model per million mapped reads) normalization (Mortazavi *et al*. 2008), that is also used in ChIPseeqer (Giannopoulou & Elemento 2011). The modified RPKM normalizes the number of mapped reads per 100 Mb of each chromosome per 10^6^ mapped reads. Thus, the normalized read number for bin *x* is *R*_*x*_ = 10^6^ × (*r*_*x*_/*N*_*i*_) × (*L*_*i*_/10^8^) where *N*_*i*_ is the total number of reads mapped onto chromosome *i* and *L*_*i*_ is the length of chromosome *i*. The number for each bin is then smoothed with a fixed width (default value 500 bp), which provides a good approximation of the real read distribution. At this stage, the wig files can be uploaded to the UCSC genome browser if the user so desires.

### DROMPA: Detecting enriched regions as potential binding sites

DROMPA scans the reference genome with a sliding window that includes contiguous bins (default value 30 bins) to identify peak regions that satisfy simultaneously the default threshold values listed below (Fig. S2 in Supporting Information):

- The enrichment p-value defined by a one-sided Wilcoxon rank-sum test between the ChIP and control is <10^−4^.
- The fold enrichment (ChIP reads per window/control reads per window) is >3.0.
- The maximum read intensity (*R*_x_) of ChIP bins in a window/the average read depth of the ChIP for the chromosome is >3.0.
- The average number of control reads per window/the average read depth of the control for the chromosome is <10.0.
- The maximum read intensity of ChIP bins in a window is >6.0.

Thresholds (i) and (ii) evaluate the significance of ChIP read enrichments against the number of control reads for each window. The Wilcoxon rank-sum test evaluates whether ChIP reads are enriched in the overall region (most of the bins) in a window. Threshold (iii) determines whether the ChIP reads mapped in a window are enriched compared with the average depth of the whole genome. When the number of control reads mapped in a window is quite large compared with the average depth of the control reads, the region is considered as a highly repetitive region, which may lead to the identification of false-positive peaks; threshold (iv) effectively filters out such peaks. Threshold (v) is useful for comparing multiple ChIP samples. The default values for these five thresholds were defined empirically.

Contiguous, significantly enriched windows are then merged, and a peak list is produced. This list can be saved as a tab-delimited text file that can be handled by a text editor or Microsoft Excel. This peak file can also be used as a bed file. DROMPA can also be used to identify broad peaks by enlarging the bin size.

### Visualization

DROMPA draws bar graphs of the mapped reads with chromosome-separated and/or whole-genome files in a pdf or png format (default, pdf format) using Cairo graphics library (http://www.cairographics.org/). These figures can display ChIP and control read distributions and ChIP/control enrichment ratios at different scales and can include genomic annotations. Because DROMPA can call peaks and visualize multiple ChIP samples simultaneously, it is a powerful tool for large-scale ChIP-seq analysis.

### Processing memory requirements

The memory required by DROMPA mainly depends on the size of the largest chromosome and the specified bin size and does not depend on the map file size. Thus, DROMPA can analyze sequence data of >100 million reads while using limited computation memory. The memory required by parse2wig depends on the map file size, but it is smaller than that used by other peak-calling programs. The total sizes of the binary and compressed wig files are much smaller than the map file.

### False discovery rate

In our published study (Deardorff *et al*. 2012), the empirical FDR was calculated as the number of control peaks/number of ChIP peaks. The same calculation is used for MACS (Zhang *et al*. 2008) and CCAT (Xu *et al*. 2010). Note that an FDR calculation would not be appropriate in the following cases: when another ChIP sample is used as a control; when the quality of the control sample is poor (e.g., many pseudobinding sites are identified); and when the ChIP sample has few peaks (e.g., <100 peaks) as shown in Peak assessment by DROMPA. To increase the universality of DROMPA, we did not use a value for the FDR as a threshold determinant.

### Performance comparison between DROMPA and other methods

MACS (Zhang *et al*. 2008), PeakSeq (Rozowsky *et al*. 2009) and Quest (Jothi *et al*. 2008) programs were executed with the default or recommended parameter settings. We supplied the ‘-optype3’ option (output peak list only) for DROMPA. For calculating memory, we used VmHWM in/proc/<PID>/status.

PeakSeq preprocesses a map file before performing peak calling, as does the combination of parse2wig and DROMPA; we therefore considered the performance of each preprocessing step separately. For Quest, we supplied a genome-table file for the peak identification process because the process took less time than when the complete genome sequence was supplied. For comparison, we used SAM-formatted map files as input for DROMPA, MACS and PeakSeq and Bowtie-formatted files for Quest, as Quest cannot handle SAM-formatted files and PeakSeq cannot handle Bowtie-formatted files.

## Analysis by DROMPA

DROMPA can include several annotation data (genes, repeats, GC contents and other BED-formatted annotation) and implement several downstream analyses. Further information of experimental design and an example workflow can be found in the Appendix S1 (Supporting Information).

## Supporting Information

**Figure S1** Memory consumption for parallel handling of multiple ChIP-control pairs.

**Figure S2** Creating wig files by parse2wig and peak calling by DROMPA.

**Figure S3** Visualization by DROMPA of the average read intensity profiles for each ChIP sample across the specified regions.

**Appendix S1** Experimental design and Procedures.

**Table S1** Disk space (GB) required for each program

**Table S2** Peak-calling results for the test data

## References

[b1] Auerbach RK, Euskirchen G, Rozowsky J, Lamarre-Vincent N, Moqtaderi Z, Lefrançois P, Struhl K, Gerstein M, Snyder M (2009). Mapping accessible chromatin regions using Sono-Seq. Proc. Natl Acad. Sci. USA.

[b2] Bailey TL, Elkan C (1994). Fitting a mixture model by expectation maximization to discover motifs in biopolymers. Proc. Int. Conf. Intell. Syst. Mol. Biol.

[b3] Bailey TL, Gribskov M (1998). Combining evidence using p-values: application to sequence homology searches. Bioinformatics.

[b4] Boeva V, Surdez D, Guillon N, Tirode F, Fejes AP, Delattre O, Barillot E (2010). De novo motif identification improves the accuracy of predicting transcription factor binding sites in ChIP-Seq data analysis. Nucleic Acids Res.

[b5] Chen Y, Negre N, Li Q (2012). Systematic evaluation of factors influencing ChIP-seq fidelity. Nat. Methods.

[b6] Chung D, Kuan PF, Li B, Sanalkumar R, Liang K, Bresnick EH, Dewey C, Keleş S (2011). Discovering transcription factor binding sites in highly repetitive regions of genomes with multi-read analysis of ChIP-Seq data. PLoS Comput. Biol.

[b7] De Piccoli G, Katou Y, Itoh T, Nakato R, Shirahige K, Labib K (2012). Replisome stability at defective DNA replication forks is independent of S phase checkpoint kinases. Mol. Cell.

[b8] Deardorff MA, Bando M, Nakato R (2012). HDAC8 mutations in Cornelia de Lange syndrome affect the cohesin acetylation cycle. Nature.

[b9] Dohm JC, Lottaz C, Borodina T, Himmelbauer H (2008). Substantial biases in ultra-short read data sets from high-throughput DNA sequencing. Nucleic Acids Res.

[b10] Enervald E, Lindgren E, Katou Y, Shirahige K, Strom L (2013). Importance of poleta for damage-induced cohesion reveals differential regulation of cohesion establishment at the break site and genome-wide. PLoS Genet.

[b11] Ernst J, Kheradpour P, Mikkelsen TS, Shoresh N, Ward LD, Epstein CB, Zhang X, Wang L, Issner R, Coyne M, Ku M, Durham T, Kellis M, Bernstein BE (2011). Mapping and analysis of chromatin state dynamics in nine human cell types. Nature.

[b12] Giannopoulou EG, Elemento O (2011). An integrated ChIP-seq analysis platform with customizable workflows. BMC Bioinformatics.

[b13] Gotoh O (2008). A space-efficient and accurate method for mapping and aligning cDNA sequences onto genomic sequence. Nucleic Acids Res.

[b14] Heintzman ND, Hon GC, Hawkins RD (2009). Histone modifications at human enhancers reflect global cell-type-specific gene expression. Nature.

[b15] Hu B, Itoh T, Mishra A, Katoh Y, Chan KL, Upcher W, Godlee C, Roig MB, Shirahige K, Nasmyth K (2011). ATP hydrolysis is required for relocating cohesin from sites occupied by its Scc2/4 loading complex. Curr. Biol.

[b16] Jothi R, Cuddapah S, Barski A, Cui K, Zhao K (2008). Genome-wide identification of in vivo protein-DNA binding sites from ChIP-Seq data. Nucleic Acids Res.

[b17] Kegel A, Betts-Lindroos H, Kanno T, Jeppsson K, Ström L, Katou Y, Itoh T, Shirahige K, Sjögren C (2011). Chromosome length influences replication-induced topological stress. Nature.

[b18] Kozarewa I, Ning Z, Quail MA, Sanders MJ, Berriman M, Turner DJ (2009). Amplification-free Illumina sequencing-library preparation facilitates improved mapping and assembly of (G+C)-biased genomes. Nat. Methods.

[b19] Kurze A, Michie KA, Dixon SE, Mishra A, Itoh T, Khalid S, Strmecki L, Shirahige K, Haering CH, Löwe J, Nasmyth K (2011). A positively charged channel within the Smc1/Smc3 hinge required for sister chromatid cohesion. EMBO J.

[b20] Laajala TD, Raghav S, Tuomela S, Lahesmaa R, Aittokallio T, Elo LL (2009). A practical comparison of methods for detecting transcription factor binding sites in ChIP-seq experiments. BMC Genomics.

[b21] Landt SG, Marinov GK, Kundaje A (2012). ChIP-seq guidelines and practices of the ENCODE and modENCODE consortia. Genome Res.

[b22] Li Q, Brown J, Huang H, Bickel P (2011). Measuring reproducibility of high-throughput experiments. Ann. Appl. Stat.

[b23] Liu ET, Pott S, Huss M (2010). Q&A: ChIP-seq technologies and the study of gene regulation. BMC Biol.

[b24] Mishra A, Hu B, Kurze A, Beckouët F, Farcas AM, Dixon SE, Katou Y, Khalid S, Shirahige K, Nasmyth K (2010). Both interaction surfaces within cohesin's hinge domain are essential for its stable chromosomal association. Curr. Biol.

[b25] Morgulis A, Gertz EM, Schaffer AA, Agarwala R (2006). WindowMasker: window-based masker for sequenced genomes. Bioinformatics.

[b26] Mortazavi A, Williams BA, McCue K, Schaeffer L, Wold B (2008). Mapping and quantifying mammalian transcriptomes by RNA-Seq. Nat. Methods.

[b27] Park PJ (2009). ChIP-seq: advantages and challenges of a maturing technology. Nat. Rev. Genet.

[b28] Rhee HS, Pugh BF (2011). Comprehensive genome-wide protein-DNA interactions detected at single-nucleotide resolution. Cell.

[b29] Rozowsky J, Euskirchen G, Auerbach RK, Zhang ZD, Gibson T, Bjornson R, Carriero N, Snyder M, Gerstein MB (2009). PeakSeq enables systematic scoring of ChIP-seq experiments relative to controls. Nat. Biotechnol.

[b30] Tanaka S, Nakato R, Katou Y, Shirahige K, Araki H (2011). Origin association of Sld3, Sld7, and Cdc45 proteins is a key step for determination of origin-firing timing. Curr. Biol.

[b31] Tazumi A, Fukuura M, Nakato R, Kishimoto A, Takenaka T, Ogawa S, Song JH, Takahashi TS, Nakagawa T, Shirahige K, Masukata H (2012). Telomere-binding protein Taz1 controls global replication timing through its localization near late replication origins in fission yeast. Genes Dev.

[b32] Xu H, Handoko L, Wei X, Ye C, Sheng J, Wei CL, Lin F, Sung WK (2010). A signal–noise model for significance analysis of ChIP-seq with negative control. Bioinformatics.

[b33] Yamaji M, Ueda J, Hayashi K, Ohta H, Yabuta Y, Kurimoto K, Nakato R, Yamada Y, Shirahige K, Saitou M (2013). PRDM14 Ensures Naive Pluripotency through Dual Regulation of Signaling and Epigenetic Pathways in Mouse Embryonic Stem Cells. Cell Stem Cell.

[b34] Zhang Y, Liu T, Meyer CA, Eeckhoute J, Johnson DS, Bernstein BE, Nusbaum C, Myers RM, Brown M, Li W, Liu XS (2008). Model-based analysis of ChIP-Seq (MACS). Genome Biol.

