# Supplementary figures and images for: DROMPA: easy-to-handle peak calling and visualization software for the computational analysis and validation of ChIP-seq data
Source: Genes Cells. 2013 May 15;18(7):589–601. doi: 10.1111/gtc.12058 (PMC3738949; doi:10.1111/gtc.12058)

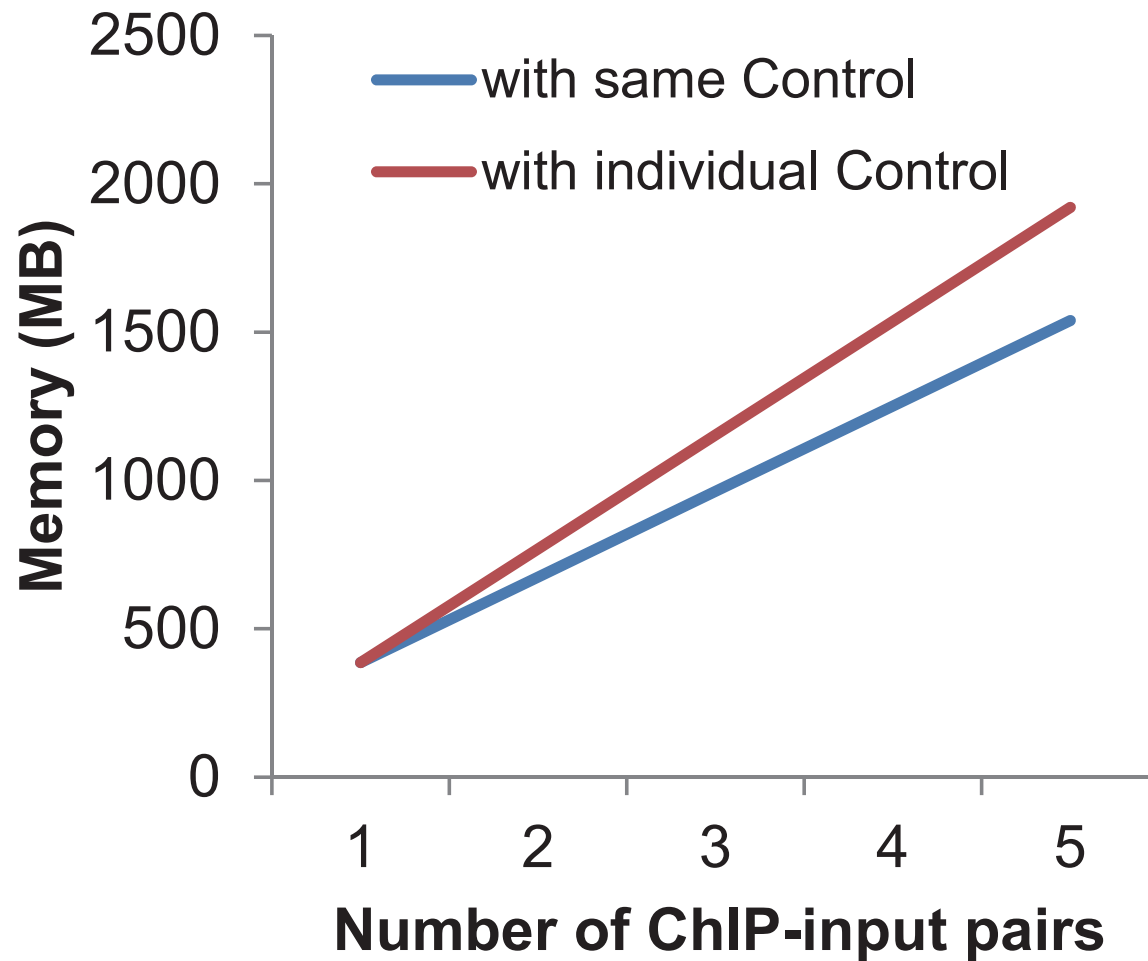

Fig.S1

Supplement: Supplementary file 1 [file gtc0018-0589-SD1.pdf]

**a**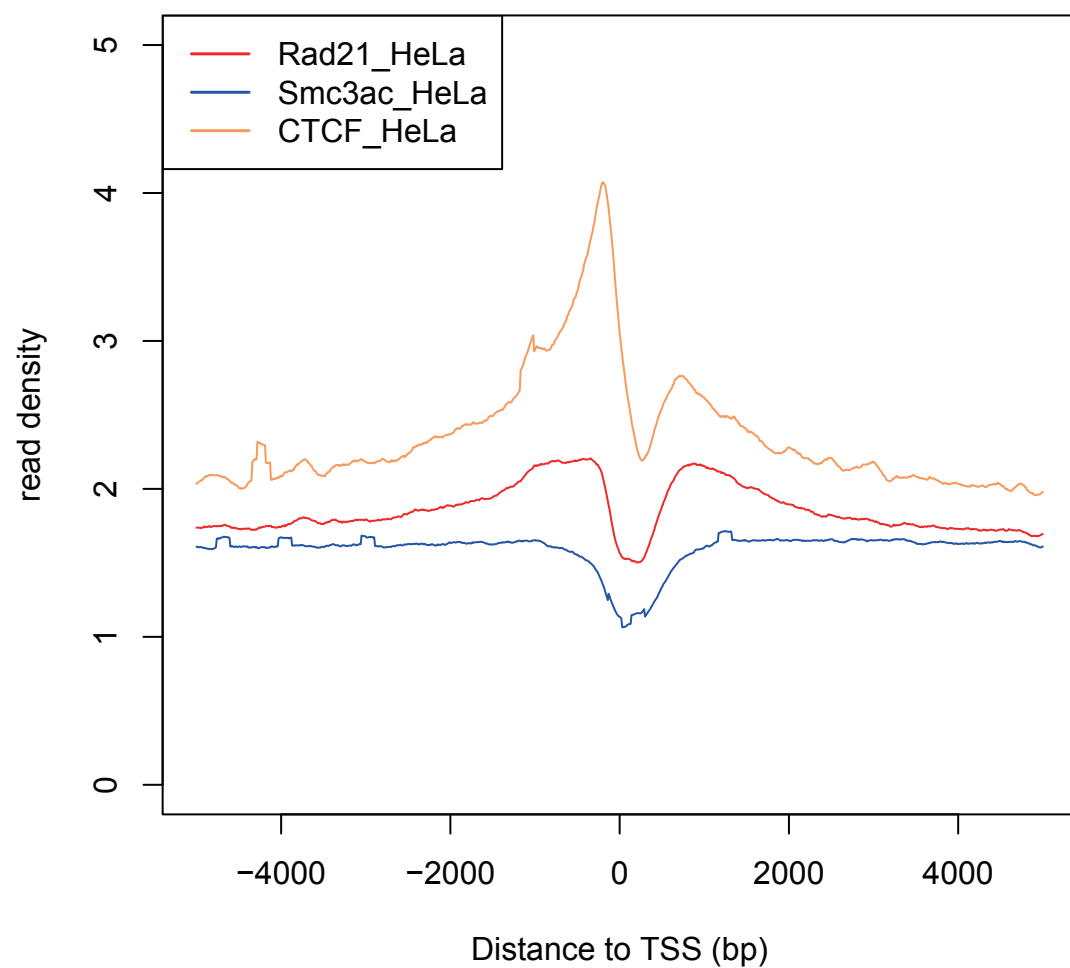**b**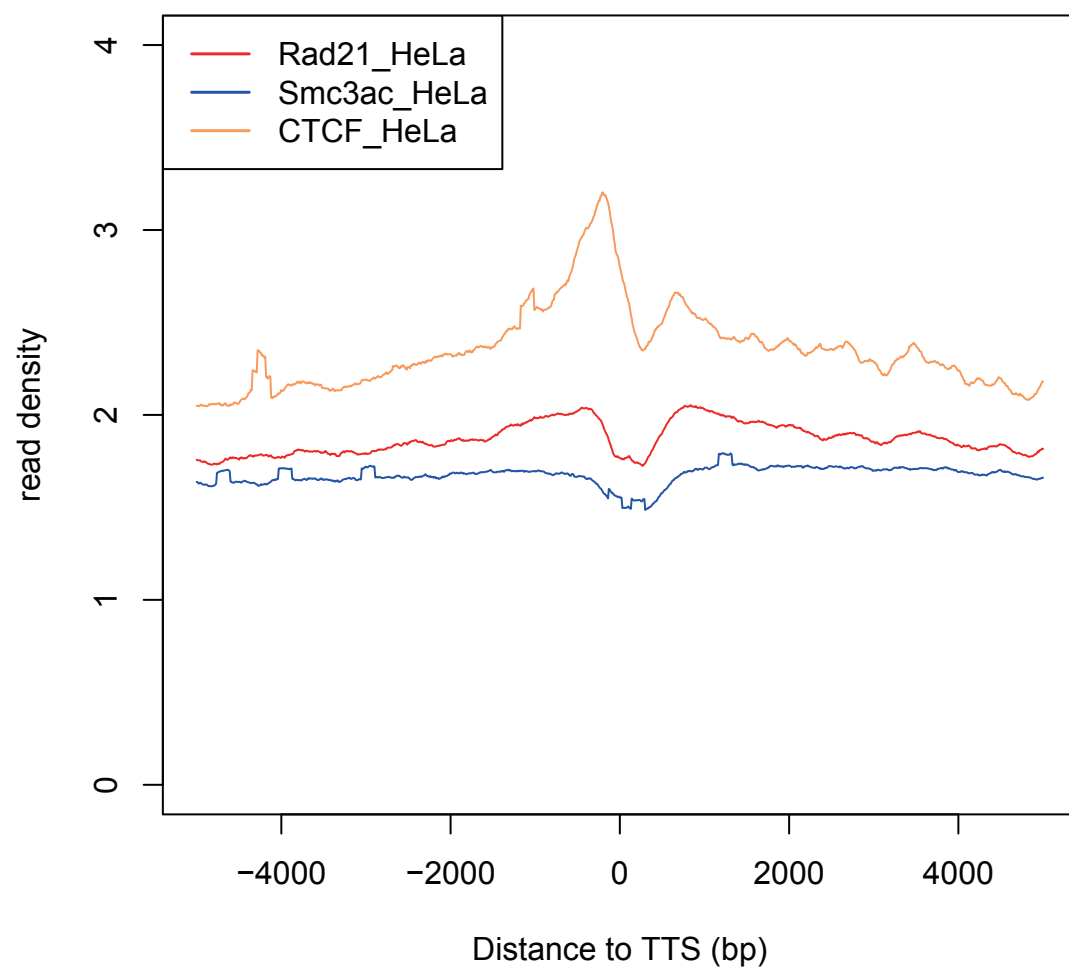**c**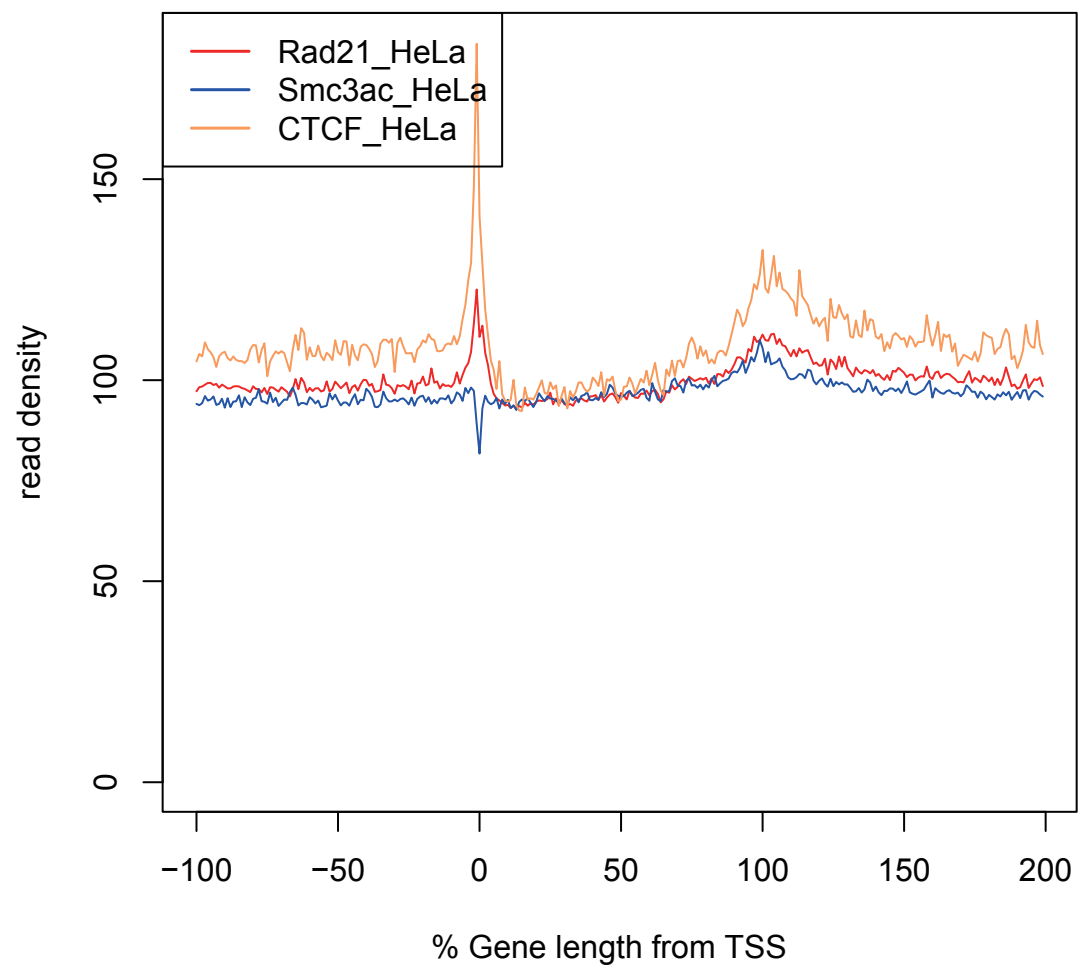**d**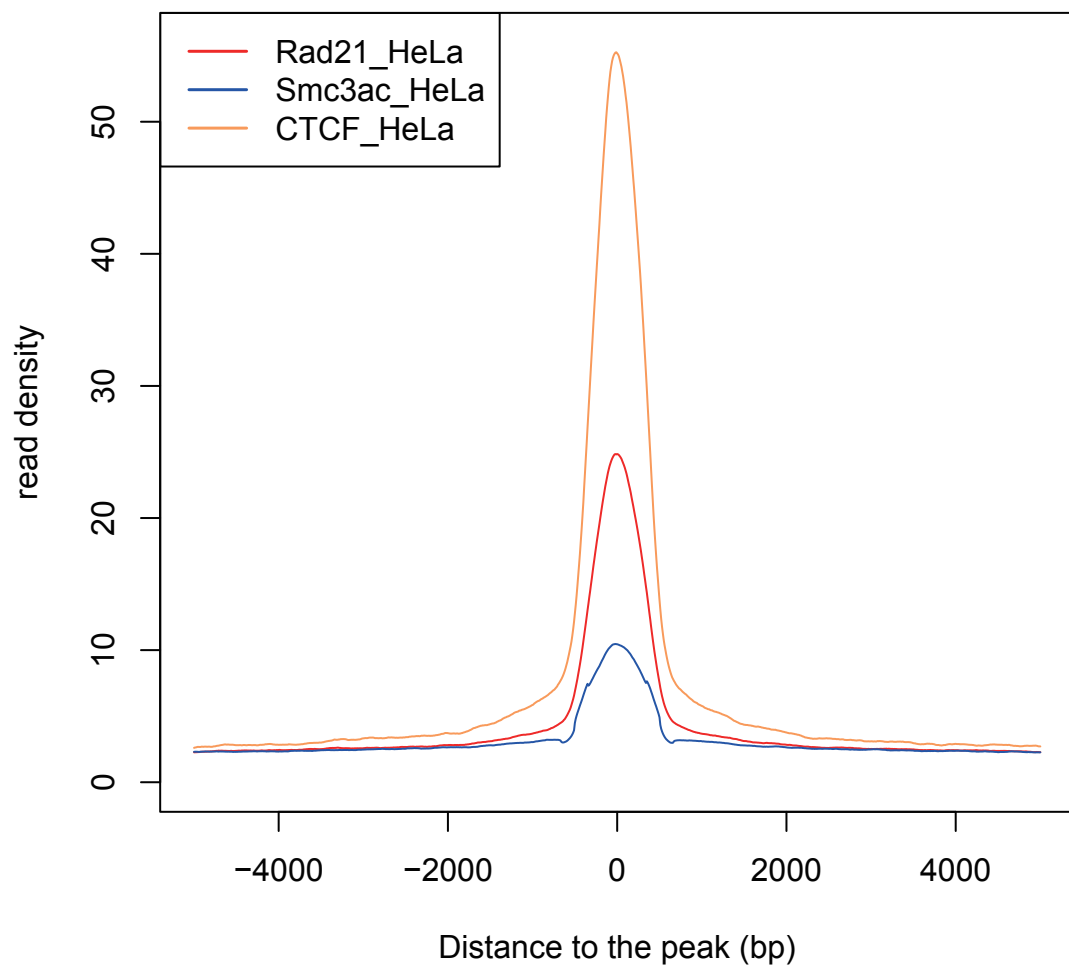

Supplement: Supplementary file 3 [file gtc0018-0589-SD3.pdf]
