# Supplementary material for: DROMPA: easy-to-handle peak calling and visualization software for the computational analysis and validation of ChIP-seq data
Source: Genes Cells. 2013 May 15;18(7):589–601. doi: 10.1111/gtc.12058 (PMC3738949; doi:10.1111/gtc.12058)
Supplement: Supplementary file 2 [file gtc0018-0589-SD2.pdf]

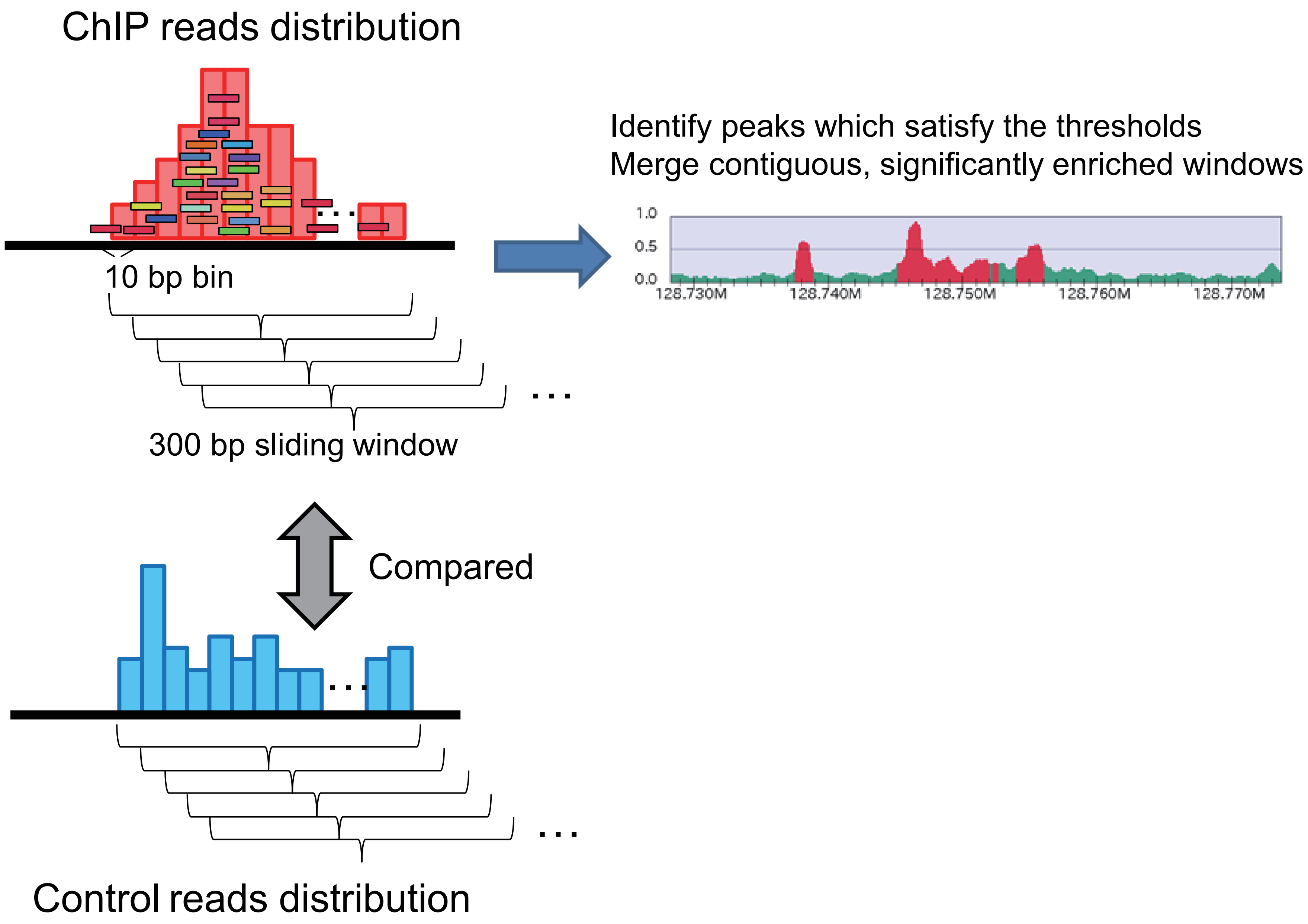
ChIP reads distribution
10 bp bin
300 bp sliding window
Identify peaks which satisfy the thresholds
Merge contiguous, significantly enriched windows
1.0
0.5
0.0
128.730M
128.740M
128.750M
128.760M
128.770M
Compared
Control reads distribution

FigS2
