# Supplementary material for: DROMPA: easy-to-handle peak calling and visualization software for the computational analysis and validation of ChIP-seq data
Source: Genes Cells. 2013 May 15;18(7):589–601. doi: 10.1111/gtc.12058 (PMC3738949; doi:10.1111/gtc.12058)
Supplement: Supplementary file 7 [file gtc0018-0589-SD7.doc]

**Supplementary Protocols**

**1) Experimental design**

**Read mapping.** For a large-scale ChIP-seq analysis, the computation time and memory requirement must be considered. When the user attempts a variety of mapping and peak-calling procedures, computation time and memory requirements increase linearly. Given this consideration, we adopted the program Bowtie (Langmead *et al.* 2009) for genome mapping because it is very fast and memory efficient. If other mapping software is used, first ensure that the files can be used by parse2wig. Several file formats for map data exist with the file format dependent on the software used to generate the file. SAMtools (<http://samtools.sourceforge.net/>) and BEDtools (<http://code.google.com/p/bedtools/>) can convert the file format to one that can be used by parse2wig. An important issue for read mapping is if multiple mapped reads are allowed. When the functional properties of repetitive sequences are of interest, it is necessary to allow multiple mapped reads (Day *et al.* 2010).

**Optimal bin size for preparing a wig file.** The optimal bin size depends on the number of reads and the binding mode of the protein[^1^](#_ENREF_1). For ChIP-seq analysis of a typical transcriptional factor, a 300-bp window (30 × 10-bp bins) is appropriate to detect binding sites. If the targeted protein is expected to bind a long DNA sequence (>100 kbp), as is the case for the binding of modified histones, a larger bin size is preferable (e.g., a 1-kbp bin).

**Threshold values for peak detection.** DROMPA uses five parameters to identify peaks (see **Detecting enriched regions as potential binding sites**). When the user wants to increase the peak-detection sensitivity or specificity, we recommend altering the parameter “maximum read intensity in the ChIP bin” (-ipm option). The results of multiple ChIP samples can be compared by applying the same value for this parameter when the signal-to-noise ratios of the samples are similar (Shao *et al.* 2012). If the signal-to-noise ratio varies from sample to sample, a different value can be set for each sample.

When the percentage of mapped reads is adequate but there are few peaks detected, there are several possible reasons. First, almost all the sequenced reads are background reads owing to one or more of the following: too small signal-to-noise ratio, inefficient immunoprecipitation of the protein or inefficient cross linking of the protein and DNA; the protein did not specifically bind DNA. Second, the protein binds DNA broadly as do several types of modified histones such as H3K27me3 and H3K36me3, and the peaks cannot be identified with the parameter set for typical transcriptional factor.

**Adding the results of other peak-calling software.** Several peak-calling programs are available that use different algorithms. Comparison of the peaks obtained by the different programs facilitates validation of the reliability of the peak-calling results. DROMPA can use the peak-calling data generated by other peak-calling programs with “-bed” option when the peak list is formatted as a BED file. BEDtools (http://code.google.com/p/bedtools/) is helpful when converting other file formats into BED format.

**Downstream analysis.** Downstream analyses are essential steps involving the characterization of the biological significance of a protein-binding profile. Several analyses are possible using DROMPA. Comparison of the binding profile for a protein with an unknown function with that of a protein with a known function may identify the function of the former. Classification of peak locations in relation to the location of a gene(s) and cis-regulatory elements on a chromosome is also an efficient way to understand the function of a protein. A peak list can be compared with genome-wide gene-expression data (e.g., microarray or RNA-seq data) to identify proteins involved during transcription. Comparison of peak intensities in different genetic backgrounds is useful when studying the effect of a mutation on the binding efficiency of a protein to its targeted DNA sequence. DROMPA has options capable of performing these types of analyses. Below we describe the procedures used previously for these downstream analyses (Deardorff *et al.* 2012). Other functional analyses (e.g., sequential motif analysis, GO analysis, and repeat analysis) are also possible (Day *et al.* 2010; Bardet *et al.* 2012) but beyond the scope of this paper.

**2) Setting up DROMPA**

**EQUIPMENT**

- Test set: ChIP-seq data for Rad21, Smc3ac, CTCF, and control for wild-type HeLa cells were obtained from the Sequence Read Archive database (http://www.ncbi.nlm.nih.gov/sra/; accession number, SRP011927).
- Reference genome sequence build hg19 from the UCSC genome browser (<http://genome.ucsc.edu/>)
- Genome-table file
- Genomic annotation files (see **BOX 1**)
- Unix-based operating system with 8 GB of RAM (16 GB preferred). We use an Ubuntu Linux OS workstation, which has a 3.0 GHz Xeon processor (4 cores, 64-bit CPU) with 24 GB of memory.
- Read mapping: we use Bowtie software (<http://bowtie-bio.sourceforge.net/index.shtml>); alternative software for read mapping is surveyed by Homer *et al.* (Li & Homer 2010).
- Preprocessing read-map files: parse2wig software
- Peak calling and visualization: DROMPA software
- R package (optional; http://www.r-project.org/)

**EQUIPMENT SETUP**

The protocols presented here use UNIX shell prompts (e.g., bash or csh). Commands are prefixed with ‘$’. “Comments” that begin with ‘#’ can be ignored.

The options and default values for the thresholds will change depending on the program version. The example provided herein uses DROMPA and parse2wig version 1.1.1. The latest versions and options are available at the DROMPA website (http://www.iam.u-tokyo.ac.jp/chromosomeinformatics/rnakato/drompa/).

**DROMPA and parse2wig setup**

DROMPA and parse2wig require the programs and libraries listed below:

- Gcc compiler (<http://gcc.gnu.org/>)
- Cairo libraries (<http://www.cairographics.org/>)
- gtk library (<http://www.gtk.org/>)
- pdftk (<http://www.pdflabs.com/tools/pdftk-the-pdf-toolkit/>)

These programs are freely available and can be installed on a Ubuntu OS workstation using the apt-get command.

Downstream analysis of the data used herein included small sections requiring an R statistical computing environment. See the R project website (<http://www.r-project.org/>) to install the R packages.

**Input and output formats for parse2wig**

Parse2wig accepts Bowtie- or SAM-formatted map files as input. Parse2wig can handle both single- or paired-end reads.

Parse2wig can output three types of wig files: a binary wig file, a compressed wig file, and an uncompressed wig file. By default, parse2wig and DROMPA use the binary wig file because it saves computation time and disk storage space. A compressed wig file requires a smaller amount of disk space than does a binary file, but its data must be decompressed for use, a process that can be time consuming. If the user wants to upload a wig file to the UCSC genome browser, a compressed wig file must be used.

**3) Procedures using test data**

**Conversion of the map file into wig files**

Here we described the protocol using uniquely mapped reads. When the user wants to use multiple mapped reads, the same protocol can be used.

DROMPA and parse2wig require a tab-delimited “genome-table” file describing the name and length of each chromosome. The chromosome name must be identical to the one used for the mapped reference genome. Here we named the genome-table file of the human genome build hg19 “genome_table-hg19.txt”.

Using the commands below, specify a bin size (here 10 bp) and a read length (here 50 bp) for the sequences contained in the map files.

$ parse2wig -i Rad21_HeLa-n3-m1-hg19.bowtie -o Rad21_HeLa-n3-m1-hg19 -binsize 10 -F3 50 -gt genome_table-hg19.txt

$ parse2wig -i Smc3ac_HeLa-n3-m1-hg19.bowtie -o Smc3ac_HeLa-n3-m1-hg19 -binsize 10 -F3 50 -gt genome_table-hg19.txt

$ parse2wig -i CTCF_HeLa-n3-m1-hg19.bowtie -o CTCF_HeLa-n3-m1-hg19 -binsize 10 -F3 50 -gt genome_table-hg19.txt

$ parse2wig -i Control_HeLa-n3-m1-hg19.bowtie -o Control_HeLa-n3-m1-hg19 -binsize 10 -F3 50 -gt genome_table-hg19.txt

The files “*.bowtie” indicate Bowtie-formatted map files. When using SAM-formatted files as input, supply “-sam” option. The output directory “parse2wigdir” (default name) is created automatically, and the chromosome-separated wig data are placed in that directory (e.g., parse2wigdir/Rad21_HeLa-n3-m1-hg19_chr1.10.bin). In the default mode, binary wig files are generated. Use the “-if1” option if compressed wig files (e.g., parse2wigdir/Rad21_HeLa-n3-m1-hg19_chr1.10.wig.gz) are desired.

**Peak calling**

When implementing DROMPA, use the prefix (before underscore, e.g., “parse2wigdir/Rad21_HeLa-n3-m1-hg19”) to specify a sample file as an input file. If the user does not need a peak-list file but wants to obtain only a pdf file, this step can be omitted.

To implement peak calling and to obtain the peak list, specify input files and output file names and implement DROMPA:

$ # specify input files

$ Rad21="parse2wigdir/Rad21_HeLa-n3-m1-hg19"

$ Smc3ac="parse2wigdir/Smc3ac_HeLa-n3-m1-hg19"

$ CTCF="parse2wigdir/CTCF_HeLa-n3-m1-hg19"

$ Control="parse2wigdir/Control_HeLa-n3-m1-hg19"

$ # call peaks

$ drompa -i $Rad21 -w $Control -p Rad21_HeLa-n3-m1-hg19-10 -optype3 -gt genome_table-hg19.txt

$ drompa -i $Smc3ac -w $Control -p Smc3ac_HeLa-n3-m1-hg19-10 -optype3 -gt genome_table-hg19.txt

$ drompa -i $CTCF -w $Control -p CTCF_HeLa-n3-m1-hg19-10 -optype3 -gt genome_table-hg19.txt

By implementing this step, the peak-list files “Rad21_HeLa-n3-m1-hg19-10.xls”, “Smc3ac_HeLa-n3-m1-hg19-10.xls” and “CTCF_HeLa-n3-m1-hg19-10.xls” are generated. Option “-optype3” produces an output peak list but not a visualization file. The default value for the peak intensity threshold is **≥** 6.0. When the user wants to set the threshold to a value **≥**10.0**,** for example, include the option “-ipm” in the command line as follows:

$ drompa -i $Rad21 -w $Control -p Rad21_HeLa-ipm10.0-n3-m1-hg19-10 -optype3 -gt genome_table-hg19.txt -ipm 10.0

**Calculation of FDR**

To calculate the FDR, obtain control peaks by replacing the ChIP samples and the Control sample as follows:

$ drompa -i $Control -w $Rad21 -p Control _Rad21_HeLa-n3-m1-hg19-10 -optype3 -gt genome_table-hg19.txt

$ drompa -i $Control -w $Smc3ac -p Control _Smc3ac_HeLa-n3-m1-hg19-10 -optype3 -gt genome_table-hg19.txt

$ drompa -i $Control -w $CTCF -p Control _CTCF_HeLa-n3-m1-hg19-10 -optype3 -gt genome_table-hg19.txt

**Pdf construction**

DROMPA has various parameters that can be used to make the desired figure. Here, we illustrate four examples of visualization, as options (A)-(D). The options can be used in any combination to make a figure. If the user does not need a pdf file but wants to obtain a peak-list file only, this step can be omitted.

**(A)** To make pdf files of ChIP-read distributions, use the following commands:

$ # specify input data and ChIP/Control pair

$ Rad21="parse2wigdir/Rad21_HeLa-n3-m1-hg19"

$ Smc3ac="parse2wigdir/Smc3ac_HeLa-n3-m1-hg19"

$ CTCF="parse2wigdir/CTCF_HeLa-n3-m1-hg19"

$Control="parse2wigdir/Control_HeLa-n3-m1-hg19"

$ s1=” -i $Rad21 -w $Control -name Rad21_HeLa”

$ s2=” -i $Smc3ac -w $Control -name Smc3ac_HeLa”

$ s3=” -i $CTCF -w $Control -name CTCF_HeLa”

$ # make pdf file

$ drompa -g_ref refFlat.txt $s1 $s2 $s3 -p ChIPseq_HeLa-n3-m1-hg19-10 -optype2 -gt genome_table-hg19.txt -LS1000

Then the pdf files (individual chromosome pdf files (e.g., ChIPseq_HeLa-n3-m1-hg19-10_chr1.pdf) and a complete genome pdf file (ChIPseq_HeLa-n3-m1-hg19-10.pdf)) are outputted. The individual chromosome pdf files are not produced if the “-rmchr” option is used.

Option “-optype2” produces output figure files without a peak list. Option “-LS1000” formats a 1000-kbp (=1-Mbp) region on one line. Option “-optype1” (default) provides both the pdf files and peak-list files. When analyzing multiple ChIP/Control pairs, the peak lists for each pair are assigned a number according to the order in which they were inputted, e.g., “ChIPseq_HeLa-n3-m1-hg19-10_1.xls”, “ChIPseq_HeLa-n3-m1-hg19-10_2.xls” and “ChIPseq_HeLa-n3-m1-hg19-10_3.xls” for Rad21, Smc3ac, and CTCF respectively.

**(B)** Visualizing specific regions with user annotations.

Specifying the “-r <file.txt>” option enables DROMPA to draw only specified regions. For example, to make a figure focusing on the MACF1 gene, make a “MACF1.txt”, tab-separated bed file and supply the option “-r MACF1.txt” as follows:

$ echo “chr1 39500000 40000000” > MACF1.txt

$ drompa -g_ref refFlat.txt $s1 $s2 $s3 -p MACF1 -optype2 -gt genome_table-hg19.txt-r MACF1.txt -LS300

To adjust the line widths according to the specified regions, the “-LS300” option is used.

When the user wants to include annotation data with BED format, e.g., enhancer regions obtained from Heintzman *et al.* (Heintzman *et al.* 2009) (here called “enhancer_hg19.txt”), use the “-bed” and “-bedname” options as follows:

$ drompa -g_ref refFlat.txt $s1 $s2 $s3 -p MACF1 -optype2 -gt genome_table-hg19.txt-r MACF1.txt -LS300 -bed enhancer_hg19.txt -bedname enhancer

The figure output obtained with this command is shown in **Figure 3**.

**(C)** Identify broad peaks

Make wig files for a bin size of 1 kbp (specify -binsize 1000 at **Conversion of the map file into wig files**) before typing the following commands.

To identify broad, enriched regions, use a 1-kbp bin size and a modified parameter set, as follows:

$ Rad21="parse2wigdir/Rad21_HeLa -n3-m1-hg19"

$ Smc3ac="parse2wigdir/Smc3ac_HeLa -n3-m1-hg19"

$ CTCF="parse2wigdir/CTCF_HeLa -n3-m1-hg19"

$Control="parse2wigdir/Control_HeLa -n3-m1-hg19"

$ s1=” -i $Rad21 -w $Control -name Rad21_HeLa”

$ s2=” -i $Smc3ac -w $Control -name Smc3ac_HeLa”

$ s3=” -i $CTCF -w $Control -name CTCF_HeLa”

$ drompa -g_ref refFlat.txt $s1 $s2 $s3 -p ChIPseq_HeLa-broad -optype2-gt genome_table-hg19.txt -LS2000 -scale_tag 25 -binsize 1000 -sw 2000 -ethre 2.0

where “-scale_tag 25” sets the maximum scale value of the *y* axis to 50, “-sw 2000” sets the smoothing width to 2 kbp, and “-ethre 2.0” sets the threshold value to >2.0-fold enrichment.

**Figure 4** shows an example of the read distribution for histone modifications H3K27me3 and H3K36me3.

**(D)** Chromosome-wide overview

Make wig files for a bin size of 100 kbp (specify -binsize 100000 in **Conversion of the map file into wig files**) before typing the following commands.

To make a chromosome-wide overview of the ChIP-seq data (**Figure 5**), specify the “-wg” option and use a larger bin size (e.g., 100 kbp). type:

$ # specify wig files and IP/Control pair

$ Rad21="parse2wigdir/Rad21_HeLa -n3-m1-hg19"

$ Smc3ac="parse2wigdir/Smc3ac_HeLa -n3-m1-hg19"

$ CTCF="parse2wigdir/CTCF_HeLa -n3-m1-hg19"

$ Control="parse2wigdir/Control_HeLa -n3-m1-hg19"

$ s1=” -i $Rad21 -w $Control -name Rad21_HeLa”

$ s2=” -i $Smc3ac -w $Control -name Smc3ac_HeLa”

$ s3=” -i $CTCF -w $Control -name CTCF_HeLa”

$ drompa -wg $s1 $s2 $s3 -p ChIPseq_HeLa-wholegenome -binsize 100000 -optype2 -gt genome_table-hg19.txt -showratio -scale_ratio 1 -graph GCcontents -gcsize 500000 -notag

For a chromosome-wide figure, we generally set the bin size to 100 kbp. **Figure 5** shows the ChIP/Control enrichment (-showratio and -notag options) with two as the maximum value for the *y* axis (-scale_ratio 1). When specifying the “-wg” option, DROMPA does not perform the significance test described in **Detecting enriched regions as potential binding sites** but simply highlights the bins containing ChIP/Control enrichments above the *y* axis (the value specified with option “-scale_ratio”) in red. The GC content for 500-kbp lengths (-graph GCcontents -gcsize 500000) is also shown (see **BOX 1** to specify the GC-content annotation).

**Downstream analysis**

These downstream procedures can be used as desired.

1. **Peak position comparison for multiple samples**

The peak files created in **Peak calling** can be compared by searching for overlapping protein-binding sites. Peak comparison is useful for the discovery of the biological function of the targeted protein and for data validation. For example, because the Rad21 peaks should overlap the CTCF peaks, comparison of the positions of the CTCF and Rad21 peaks could be used to verify both sets of data. Although we performed such a comparison with our in-house program, BEDTools also reports overlapping peaks in two bed files (e.g., intersectBED).

1. **Peak distribution analysis in relation to genes and cis-acting elements**

The classification of a peak position in relation to the location of a gene and/or a cis-acting element is useful for the discovery of a protein’s function and validation of the results. Gene-structure data can be obtained from Ensembl (http://ensembl.org/). By extracting each genomic region (i.e., upstream, downstream, exonic, intronic, or intergenic) and formatting it as a bed file, the percentage of overlapping peaks within each genomic region can be calculated by applying the same protocol as for the peak comparison of **Downstream analysis(A)**. For our works, upstream and downstream are defined as regions within 5 kb of the 5' and 3' ends of an open reading frame, respectively.

1. **Peak intensity comparison for multiple samples**

A peak-intensity comparison is optional; it is difficult to find internal sites that can be used as controls for the normalization of the peak intensities in multiple samples. However, comparison of the peak intensities among samples that were obtained using the same antibody and cell line (e.g., distribution of the same target protein under different conditions) is sometimes informative, and the differences in peak intensities roughly reflect the quantitative differences in the amounts of protein bound at each site.

DROMPA can show the average read distribution around transcription start sites (TSS), transcription termination sites (TTS), gene bodies, and specified peaks (**Supplementary Figure 3**). To do so, use the “-profile” option as follows:

$ # show read density around TSS

$ drompa -profile1 -g_ref refFlat.txt $s1 $s2 $s3 -p aroundTSS -gt genome_table-hg19.txt

$ # show read density around TTS

$ drompa -profile2 -g_ref refFlat.txt $s1 $s2 $s3 -p aroundTTS -gt genome_table-hg19.txt

$ # show read density around gene bodies

$ drompa -profile3 -g_ref refFlat.txt $s1 $s2 $s3 -p aroundgene -gt genome_table-hg19.txt

$ # show read density around peak lists

$ drompa -profile4 -g_ref refFlat.txt $s1 $s2 $s3 -p aroundSmc3ac -gt genome_table-hg19.txt

DROMPA outputs a script for R. To use the R command type:

$ R --vanilla < aroundTSS.R

$ R --vanilla < aroundTTS.R

$ R --vanilla < aroundgene.R

$ R --vanilla < aroundSmc3ac.R

The pdf files “aroundTSS.pdf”, “aroundTTS.pdf”, “aroundgene.pdf”, and “aroundSmc3ac.pdf” are outputted.

**Figure S1. Memory consumption for parallel handling of multiple ChIP-control pairs.**

The total memory (MB) used versus the number of ChIP-control pairs. When using the same control sample with all ChIP samples (blue line), DROMPA required less memory than that when using a different control sample with each ChIP sample (red line).

**Figure S2. Creating wig files by parse2wig and peak calling by DROMPA.**

DROMPA scans the ChIP read distributions in the wig files generated by parse2wig with a sliding window that includes contiguous bins (default value, 30 bins = 300-bp window). Significantly enriched windows are identified on the basis of threshold values for five parameters. Contiguous windows are merged into one peak region, and the series of peaks are identified. The identified peaks are highlighted in red, and non-enriched regions are colored green.

**Figure S3. Visualization by DROMPA of the average read intensity profiles for each ChIP sample across the specified regions.**

(**a**) Transcription start sites (TSS)-centered plots. (**b**) Transcription termination sites (TTS)-centered plots. (**c**) Percent gene length from the TSS. (**d**) Peak-centered plots.

**References**

Bardet, A.F., He, Q., Zeitlinger, J. & Stark, A. (2012) A computational pipeline for comparative ChIP-seq analyses. *Nat Protoc* **7**, 45-61.

Day, D.S., Luquette, L.J., Park, P.J. & Kharchenko, P.V. (2010) Estimating enrichment of repetitive elements from high-throughput sequence data. *Genome Biol* **11**, R69.

Deardorff, M.A., Bando, M., Nakato, R. *et al.* (2012) HDAC8 mutations in Cornelia de Lange syndrome affect the cohesin acetylation cycle. *Nature* **489**, 313-317.

Heintzman, N.D., Hon, G.C., Hawkins, R.D. *et al.* (2009) Histone modifications at human enhancers reflect global cell-type-specific gene expression. *Nature* **459**, 108-112.

Langmead, B., Trapnell, C., Pop, M. & Salzberg, S.L. (2009) Ultrafast and memory-efficient alignment of short DNA sequences to the human genome. *Genome Biol* **10**, R25.

Li, H. & Homer, N. (2010) A survey of sequence alignment algorithms for next-generation sequencing. *Brief Bioinform* **11**, 473-483.

Shao, Z., Zhang, Y., Yuan, G.C., Orkin, S.H. & Waxman, D.J. (2012) MAnorm: a robust model for quantitative comparison of ChIP-Seq data sets. *Genome Biol* **13**, R16.

**BOX 1) Annotation data for DROMPA**

DROMPA accepts annotation data from the publicly accessible websites listed below. These annotation files can also be downloaded from the DROMPA website (http://www.iam.u-tokyo.ac.jp/chromosomeinformatics/rnakato/drompa/). See the website for more information.

**(i) Gene annotation data (RefSeq)**

DROMPA accepts RefSeq gene annotation (“refFlat.txt”) obtained from the UCSC genome website (http://genome.ucsc.edu/). Download refFlat.txt, and supply option “-g_ref” as follows:

$ drompa -g_ref refFlat.txt $s1 $s2 $s3 -p ChIPseq_HeLa -optype2 -gt genome_table-hg19.txt -LS1000

**(ii) Gene data (Ensembl)**

Supply option “-g_ens” to include Ensembl gene annotation data (called “Ensemblgene.txt” here).

$ drompa -g_ens Ensemblgene.txt $s1 $s2 $s3 -p ChIPseq_HeLa -optype2 -gt genome_table-hg19.txt -LS1000

This annotation data was obtained from BioMart at the Ensembl website (<http://ensembl.org/>), and modified for DROMPA.

**(iii) Genomic annotation data (*S. cerevisiae*)**

The genomic annotation data for *S. cerevisiae* can be obtained from the *Saccharomyces* Genome Database (SGD, <http://www.yeastgenome.org/>). Download “SGD_features.tab”, and supply the option “-g_scer” as follows:

$ drompa -g_scer SGD_features.tab $s1 $s2 $s3 -p ChIPseq_Scer -optype2 -gt genome_table-scer.txt -LS50

**(iv) Gene annotation data (*S. pombe*)**

For the gene annotation data for *S. pombe,* download a GFT-formatted file (e.g., “schizosaccharomyces_pombe.EF1.62.gtf”) from Ensembl and supply the option “-g_spom” as follows:

$ drompa -g_spom schizosaccharomyces_pombe.EF1.62.gtf $s1 $s2 $s3 -p ChIPseq_Spom -optype2 -gt genome_table-spom.txt -LS50

**(iv) Replication origin data (*S. cerevisiae* and *S. pombe*)**

DROMPA can visualize DNA replication origin data available for yeast. The annotation data can be obtained from OriDB (<http://www.oridb.org/>). Download the origin list (called “Orilist.txt” here), and supply the option “-ars” as follows:

$ drompa -g_sgd SGD_features.tab -ars Orilist.txt $s1 $s2 $s3 -p ChIPseq_Scer -optype2 -gt genome_table-scer.txt -LS50

**(v) Repeat data (RepBase)**

RepBase data can be obtained from Table Browser of the UCSC genome website (http://genome.ucsc.edu/). Download the data (called “repeat.txt” here), and specify the option “-repeat” as follows:

$ drompa -repeat repeat.txt $s1 $s2 $s3 -p ChIPseq_HeLa -optype2 -gt genome_table-hg19.txt -LS1000

**(vi) GC contents**

DROMPA can incorporate bed-formatted GC content files as input. Download the compressed GCcontents data (called “gccontents10k.tar.gz” here), and specify a directory for it as follows:

$ tar zxvf gccontents10k.tar.gz

$ drompa -graph gccontents10k -gcsize 10000 $s1 $s2 $s3 -p ChIPseq_HeLa -optype2 -gt genome_table-hg19.txt -LS1000

To supply an arbitrary window size, the DROMPA website provides a program “GCcount.pl” to create these files from a FASTA-formatted file.

**(vii) Other annotations in bed file**

DROMPA can incorporate other annotation files in bed format (e.g., important regions identified by preliminary examination) into a figure using the “-bed” option. When the user wants to show the annotation file “annotation.bed” named “my_annotation”, type:

$ drompa $s1 $s2 $s3 -p ChIPseq_HeLa -optype2 -gt genome_table-hg19.txt -LS1000 -bed annotation.bed -bedname my_annotation

**Table S1. Disk space (GB) required for each program.**

|  | Rad21 | Smc3ac | CTCF | Control |
| --- | --- | --- | --- | --- |
| Read file (csfastq) | 13.9 | 22.1 | 7.8 | 20.3 |
| Map file (SAM) | 12.4 | 19.7 | 7.0 | 18.0 |
| PeakSeq preprocess data | 3.4 | 3.4 | 3.4 | 4.6 |
| Wig files (binary) | 1.2 | 1.2 | 1.2 | 1.2 |
| Wig files (compressed) | 0.6 | 0.7 | 0.4 | 0.6 |

**Table S2.** Peak-calling results for the test data.

| Antibody | Number of ChIP peaks | Number of control peaks | FDR (%) |
| --- | --- | --- | --- |
| Rad21 | 34,631 | 59 | 0.17 |
| Smc3ac | 5,456 | 10 | 0.18 |
| CTCF | 56,578 | 233 | 0.41 |
